# PATTERNFINDER: combined analysis of DNA regulatory sequences and double-helix stability

**DOI:** 10.1186/1471-2105-5-134

**Published:** 2004-09-21

**Authors:** Yanlin Huang, David Kowalski

**Affiliations:** 1 Cancer Genetics Department, Roswell Park Cancer Institute, Buffalo, NY 14263, USA; 2 Microsoft Corp., Redmond, WA 98052, USA

## Abstract

**Background:**

Regulatory regions that function in DNA replication and gene transcription contain specific sequences that bind proteins as well as less-specific sequences in which the double helix is often easy to unwind. Progress towards predicting and characterizing regulatory regions could be accelerated by computer programs that perform a combined analysis of specific sequences and DNA unwinding properties.

**Results:**

Here we present PATTERNFINDER, a web server that searches DNA sequences for matches to specific or flexible patterns, and analyzes DNA helical stability. A batch mode of the program generates a tabular map of matches to multiple, different patterns. Regions flanking pattern matches can be targeted for helical stability analysis to identify sequences with a minimum free energy for DNA unwinding. As an example application, we analyzed a regulatory region of the human c-myc proto-oncogene consisting of a single-strand-specific protein binding site within a DNA region that unwinds*in vivo*. The predicted region of minimal helical stability overlapped both the protein binding site and the unwound DNA region identified experimentally.

**Conclusions:**

The PATTERNFINDER web server permits localization of known functional elements or landmarks in DNA sequences as well as prediction of potential new elements. Batch analysis of multiple patterns facilitates the annotation of DNA regulatory regions. Identifying specific pattern matches linked to DNA with low helical stability is useful in characterizing regulatory regions for transcription, replication and other processes and may predict functional DNA unwinding elements.

PATTERNFINDER can be accessed freely at:

## Background

Regulatory regions in DNA are comprised of multiple functional elements that act in *cis *to control transcription, replication and other biological processes. Types of *cis*-acting DNA elements include unique sequences that bind specific proteins, spacer elements that provide a proper distance for interaction among protein binding sites, and structural elements that determine the flexibility of the DNA sequence. The structure of DNA is crucial to genetic regulation. The double helix needs to be locally unwound at start sites for DNA replication [1]. Additionally, DNA unwinding, flexibility and topology are important in regulating gene transcription and other processes [2].

Little is known about functional elements in most gene regulatory regions. Annotation of regulatory regions lags far behind the annotation of features of coding regions in DNA sequence databases. Best characterized are specific sequences that bind known proteins *in vitro *and whose functional importance has been determined by mutational analysis *in vivo*. Not as well characterized, but also important, are less-specific sequences that play roles in determining DNA structure and flexibility. DNA in certain regulatory sequences has a low helical stability as revealed by hypersensitivity to single-strand-specific nucleases and by stable DNA unwinding seen in plasmid topoisomers [3,4]. Helical stability minima computed using thermodynamic properties of nearest-neighbor nucleotides correctly predict the locations and hierarchy of the nuclease-hypersensitive sites [5]. Mutational analysis has revealed a functional region of low helical stability, called a DNA unwinding element, in replication origins in several species [6-9]. Some regulatory sequences with a low helical stability have been demonstrated to adopt an unwound DNA structure inside cells [10,11]. Low helical stability of DNA is of general importance since it is a feature of regulatory sequences involved in a variety of cellular processes including gene transcription, replication, nuclear matrix attachment and DNA repeat instability [6,10,12-14].

Progress towards characterizing and predicting regulatory regions could be accelerated by the availability of computer programs that perform a combined analysis of DNA sequence patterns and DNA unwinding properties. Here we present PATTERNFINDER, an easy to use web server that combines the search for specific or flexible sequence patterns with an analysis of DNA helical stability. The output of a DNA pattern search can be linked to THERMOCALC, a new program which ranks the helical stabilities of pattern matches, and an enhanced version of WEB-THERMODYN [15], which profiles the helical stability of individual matches and finds the most easily-unwound sequence. Below is information on using the PATTERNFINDER web server as well as an example analysis of the multi-functional regulatory region of a human proto-oncogene, c-myc.

## Results

### PATTERNFINDER Web Server

Web servers offer advantages to the user including platform-independent software that requires no installation and a friendly browser interface for data entry and display. The PATTERNFINDER web server searches DNA sequences for matches to specific or flexible patterns, and analyzes DNA helical stability within or flanking the matches. Examples of specific patterns include sequences known to be recognized by particular proteins or enzymes, or sequences complementary to oligonucleotide primers. Flexible patterns include, for example, consensus sequences containing ambiguous nucleotides and unspecified nucleotides (N's), or multiple specific patterns separated by a variable number of N's, or simple sequences that are repeated a variable number of times. The DNA pattern used in the search can be comprised of A, G, C, T, ambiguous nucleotides (IUPAC-IUB nomenclature), and N's. Tables listing abbreviations of ambiguous nucleotides (e.g., W = A or T) and annotations for patterns using N's and sequence repeats are provided on the home page of the web server [16]. Mismatches are not permitted. Including N's in a pattern query is useful for retrieving sequences adjacent to a pattern match or known landmark (e.g., N{100}GAATTC = 100 bp sequence 5' to GAATTC) for further analysis. N's can also be used to fix or vary spacing between patterns, as indicated on the home page. The maximum pattern span entry must include the total number of nucleotides in the pattern, including repeats and N's (span = 106 in the example above). The span entry can not exceed the total length of the DNA sequence or region analyzed.

Users input the name of the DNA molecule, the shape (linear or circular) and the nucleotide sequence. The sequence can be uploaded from a computer file (≤40 kb) in a various user-selected formats (ASCII text, Genbank, Fasta). Alternatively, the sequence can be pasted (≤30 kb) or typed into the DNA Sequence Query window of the browser. Acceptable characters are A, G, C, and T. Integers used for sequence numbering are permitted. Under the "Analysis Parameters" the user can select to search the whole DNA molecule or a part of the molecule from one position to another. Both DNA strands are searched by default in order to find matches to asymmetric patterns. If desired, the search can be restricted to only the upper (+, input) or lower (-, complementary) strand by selecting the appropriate checkbox.

The output displays the molecule name, size in bp, and size of the region analyzed, and the DNA sequence of the upper and lower strands. Note that all DNA sequences in the output are displayed 5'->3' regardless of strand. This is useful for creating sequence alignments. The total number of hits and the pattern query are displayed. Tabulated are the position, strand (+ or -) and sequence of all pattern matches. The data are also output to an ASCII text file for printing, archiving and for any further processing (e.g., in a spreadsheet).

A BATCH version of PATTERNFINDER is included to search simultaneously for multiple patterns in a DNA sequence. Entries are pasted or typed into the BATCH query window, one per line. Entries take the form of "Pattern name, Pattern expression, Maximum span". A table of pattern names and expressions that were entered is included in the output. The tabular output of hits is similar to that for PATTERNFINDER (above) except that it also includes the names of patterns that are hit as well as those that are not hit. The ASCII output provides a tabular map of the positions and strands of different pattern matches found in the DNA sequence. In addition to flexible sequence patterns, BATCH lists can include a variety of specific DNA elements and landmarks such as protein binding sequences, restriction sites and oligonucleotide primers.

Low helical stability of DNA is often a property of regulatory sequences [3,5,6]. A unique feature of PATTERNFINDER is that the output can serve as input for analysis of double-helix stability within or flanking the pattern matches. Selecting the appropriate checkbox on the input page enables THERMOCALC or WEB-THERMODYN to process the output of the pattern search. THERMOCALC is a new program that analyzes multiple DNA sequences in a user-selected, fixed window and ranks the free energy values. WEB-THERMODYN performs a sliding-window analysis of a DNA sequence to profile the helical stability and to identify the most easily-unwound sequence (free energy minimum). The utility of the published WEB-THERMODYN program [15] has been enhanced. The results of a PATTERNFINDER sequence search are directly output into an new input page for WEB-THERMODYN that accomodates multiple sequence entries, permiting the facile and rapid analysis of multiple pattern hits. The algorithm used to calculate free energy (ΔG) from thermodynamic parameters of nearest-neighbor nucleotides is described on the web server [17]. Briefly, the standard entropy and enthalpy values for each of the ten possible nearest-neighbor nucleotide interactions present in a DNA sequence are individually summed and then used to calculate the free energy using the equations previously described [9]. The WEB-THERMODYN program has also been upgraded to include the unified thermodynamic data set of SantaLucia [18]. This is now the current default data set since it represents a consensus agreement among six independent data sets and has been found to rank the free energies of DNA sequences even more accurately than the previous data set [18]. A drop down menu permits selection of the current default or the previous data set.

Default values for temperature and salt concentration [3] are present on the input page and these values can be altered by the user if desired. The input value for the start position is used by THERMOCALC to calculate ΔG from that position to the end of the sequence hit. Input values for Start Position, Step Size, Window Size and Number of Markers at minimum energy windows are used by WEB-THERMODYN, which also provides links to the DNA sequences at energy minima and a graphical profile of helical stability. Enabling WEB-THERMODYN also permits further adjustment or variations of the parameters for helical stability analysis on the PATTERNFINDER output page.

PATTERNFINDER was designed to be fast and user friendly. No password restriction or registration is required. All entries are error checked before processing and, if errors exist, the user is prompted with specific suggestions to correct the entries. The output is returned directly to the browser in real time, as opposed to a return via e-mail at a later time. The home page of the web server provides a DEMO link [19] containing sequence and pattern files relevant to the application that follows.

### PATTERNFINDER analysis of the regulatory region of the human c-myc gene

**Figure 1:**
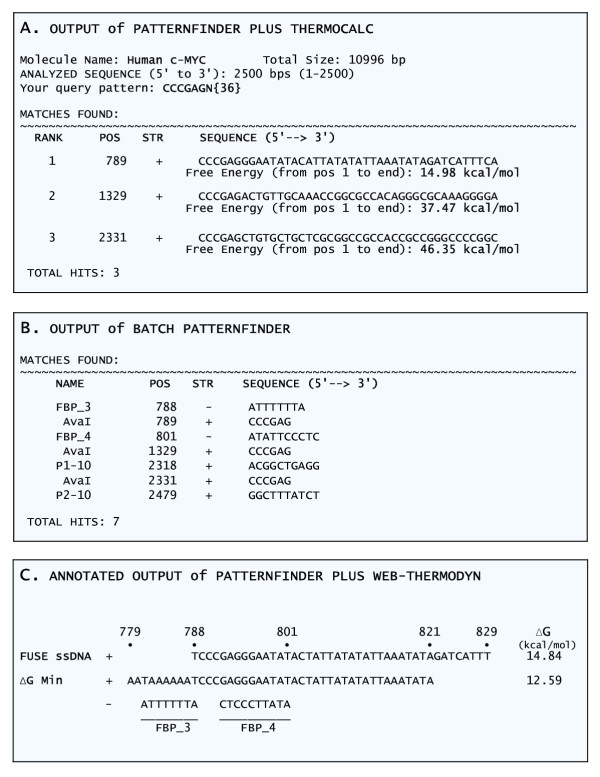
Examples of analysis of the human c-myc gene regulatory region using PATTERNFINDER in conjunction with THERMOCALC (A), in BATCH mode (B), and with WEB-THERMODYN (C) [see text].

The 5' regulatory region of the c-myc gene resides in the first 2500 bp of an ~11 kb DNA sequence. The sequence contains multiple elements that regulate transcription and replication [10,12], but the locations of the DNA elements are not yet annotated in public databases. The far upstream sequence element (FUSE) acts in *cis *to stimulate c-myc promoter activity and a 42 bp sequence becomes single-stranded *in vivo *(HeLa cells) when the gene is actively transcribed [10]. The single-stranded region is located primarily 3' to a specific *Ava I *restriction enzyme site. PATTERNFINDER was used to search for specific *Ava I *sites and any ("n") 36 bp sequences in a 42 bp segment (pattern: CCCGAGn{36}; span = 42) with THERMOCALC enabled to rank the DNA helical stabilities of the segments. The search was restricted to the regulatory region from positions 1 to 2500 (entered under the "Analysis Parameters"). The output is shown in Fig. 1A. The locations and strands of three *Ava I *sites found and the helical stability ranks (free energy, kcal/mol) of the 42 bp segments are displayed. The site with rank 1 has the lowest free energy and begins at position 789 on the + strand. As shown below, this predicted region of low helical stability contains the single-stranded DNA region identified experimentally in the FUSE.

To map the locations of other DNA elements in the 5' regulatory region, the BATCH version of PATTERNFINDER was used to search simultaneously for multiple, specific sequences. The patterns included two separate sequences that interact with the FUSE binding protein (FBP) in domains 3 and 4 [20], the *Ava I *site, and sequences of the -10 regions at the P1 and P2 promoters for c-myc transcription [10]. The BATCH output in Fig. 1B shows the precise locations and strands of the DNA elements in relation to the *Ava I *sites, providing an informative tabular map of functional elements and landmarks in the regulatory region.

In conjunction with PATTERNFINDER, WEB-THERMODYN was used to profile the DNA helical stability of all 42 bp windows in a ~400 bp region containing the binding site for FBP domain 3 at the center. The pattern query used was n{200}TAAAAAATn{200} and the span = 408. This query finds the complementary sequence (+ strand) to the FBP_3 site (ATTTTTTA, - strand) and retrieves the 200 nt sequences 5' and 3' for the WEB-THERMODYN analysis (Step Size = 1, Window Size = 42, Number of Markers = 1[energy minima]). Fig. 1C shows that the sequence with the minimum free energy (ΔG Min) in the 408 bp region analyzed overlaps most of the single-stranded DNA region detected experimentally at FUSE *in vivo *(FUSE ssDNA), reflecting a reduced helical stability intrinsic to DNA at FUSE. A separate WEB-THERMODYN analysis of the entire 2500 bp regulatory region identified the identical sequence as the ΔG Min, indicating that the low helical stability at FUSE is unique within the entire 5' regulatory region. The location of the ΔG Min sequence (+ strand shown in Fig. 1C) also overlaps the single-stranded DNA binding sites for FBP_3 and FBP_4 present in the -strand. The values of ΔG Min and ΔG FUSE ssDNA are well below the average ΔG (29.44 kcal/mol) for all 42 bp windows for the entire 2500 bp region. The predicted DNA sequence with the lowest helical stability in the 5' regulatory region contains the single-stranded DNA region detected experimentally at FUSE [10].

## Discussion

The PATTERNFINDER web server provides a convenient means to search DNA regulatory regions for specific or flexible sequence patterns and to identify flanking sequences that are easy to unwind. Specific sequence searches permit localization of known protein binding sites and sequence landmarks such as restriction sites and oligonucleotide primers. The capacity to perform BATCH analysis of multiple patterns and generate accurate tabular maps facilitates the annotation of DNA regulatory regions. The latter lags far behind the annotation of protein coding regions in public sequence databases, and individual laboratories must usually draw on their own annotation resources in order to design experiments. The utility of PATTERNFINDER in annotation of known DNA elements can facilitate the design of experiments to further characterize regulatory regions. New DNA elements with potential functions can be predicted using flexible patterns such as consensus sequences containing ambiguous nucleotides and N's as well as using multiple motifs with fixed or variable spacing. Sequences found are displayed 5'->3' regardless of the DNA strand which is useful for creating sequence alignments for position weight matrices and for identifying evolutionary conserved sequences. More sophisticated pattern match and discovery methods exist ([21], and references therein) and advanced versions of PATTERNFINDER that permit base mismatches and utilize weight matrices are under development. A strength of the current version of PATTERNFINDER is the capacity to identify pattern matches that are linked to DNA sequences of low helical stability. Such a combined search for two different types of DNA elements can lead to predictions of greater specificity than can be obtained by searching for either element alone and may help predict functional DNA unwinding elements associated with specific protein binding sites. The availability of PATTERNFINDER will hopefully stimulate experiments to verify the biological function of predicted DNA elements.

The ability to find DNA sequences and to target the flanking regions for helical stability analysis is a unique feature of PATTERNFINDER. This feature is useful in characterizing regulatory regions for gene transcription and DNA replication which require specific protein-binding sequences as well as less-specific flanking sequences in which the double helix is easy to unwind. Helical stability is ranked by the free energy value beginning with the most easily-unwound sequence by enabling a new program, THERMOCALC. Helical stability is profiled to identify free energy minima by enabling an enhanced version of the previously described WEB-THERMODYN program [15]. As shown in the Results for the transcription regulatory region of the human proto-oncogene, c-myc, the predicted region of minimum helical stability overlaps significantly the FUSE single-stranded DNA region identified experimentally *in vivo *[10]. The predicted region of minimum helical stability also overlaps the protein binding sites for FBP domains 3 and 4 [20]. FUSE is an example of a DNA unwinding element that appears to function through both intrinsic helical instability and binding a protein, FBP, a single-strand-specific DNA binding protein with a non-processive helicase activity [10,22].

Our analysis of helical stability is general and makes no assumptions about the DNA unwinding mechanism. DNA opening at FUSE has been proposed to be induced by torsional stress generated by transcription of the c-myc gene [10]. Consistent with a role for torsional stress, an independent computer analysis predicted a high probability of DNA opening at FUSE at specific levels of negative supercoiling [23]. WEB-THERMODYN analysis predicted an overlapping sequence as the helical stability minimum of the c-myc regulatory region (Fig. 1C). When the DNA opening mechanism is initiated by torsional stress alone, helical stability analysis predicts the site that corresponds to the DNA sequence of lowest helical stability identified experimentally in negatively supercoiled DNA *in vitro *[3,5]. When the DNA opening mechanism requires prior binding of proteins to duplex DNA, as is the case for replication origins (see below), functional DNA opening in a cell can occur at a low helical stability sequence that does not necessarily correspond to the lowest-stability site that opens in negatively supercoiled DNA *in vitro *[24]. In the latter case, consideration of specific protein binding sequences in addition to the helical stability is required, as is done in PATTERNFINDER with THERMOCALC or WEB-THERMODYN enabled. Our computer analysis described here and that of He et al. [23] provide useful and complementary information about DNA regulatory sequences, and both analyses could be employed to take full advantage of their individual strengths.

PATTERNFINDER will also have direct application in the further characterization of DNA replication origins, which are comprised of origin recognition elements, less-specific sequences including a DNA unwinding element, and additional elements such as transcription factor binding sites [1,6,7,11,25,26]. Interestingly, the transcription regulatory region of the c-myc gene also contains a replication origin, and a low helical stability region in the vicinity of FUSE has been suggested to function as a DNA unwinding element that facilitates replication initiation [12]. A functional role in initiation of c-myc replication for FBP, the single-strand DNA binding protein that binds FUSE and has a non-processive helicase activity [10,22], is not known. The function of several well-characterized replication origins requires an initiator protein complex that recognizes double-stranded DNA [1,11,25]. Also important is a DNA unwinding element [7], which must be properly spaced and oriented relative to the sequences that bind initiator proteins in certain origins [6,9]. PATTERNFINDER is capable of searching DNA sequences for all of the features of replication origins: specific sequences, less-specific sequences including those that are easily-unwound, and proper orientation and spacing between multiple types of sequences. Finally, PATTERNFINDER is likely to have applications in studying processes in addition to replication and transcription, such as nuclear matrix attachment and DNA repeat instability, which also involve specific DNA sequences and a region of low helical stability [13,14].

## Conclusions

The PATTERNFINDER web server will be useful in annotating, predicting and characterizing regulatory regions for replication, transcription and other processes that require specific or less specific sequences recognized by particular proteins and additional sequences in which the double-helix is unstable and functions in localized DNA unwinding.

## Methods

The PATTERNFINDER program was written in Practical Extraction and Report Language (PERL) and HyperText Markup Language (HTML) and uses the Common Gateway Interface (CGI) for input and output to a web browser. The DNA sequence of the human c-myc gene was obtained from GenBank (accession number: X00364). The average ΔG for all windows was determined by analysis of the WEB-THERMODYN output using spreadsheet software.

## Authors' Contributions

YH wrote the program code for PATTERNFINDER and established a web site. DK served as scientific advisor and participated in the program design and development.

## References

[B1] DePamphilis ML, DePamphilis ML (1996). Origins of DNA Replication. DNA Replication in Eukaryotic Cells.

[B2] Sinden RR (1994). DNA Structure and Function.

[B3] Kowalski D, Natale DA, Eddy MJ (1988). Stable DNA unwinding, not "breathing," accounts for single-strand-specific nuclease hypersensitivity of specific A+T-rich sequences. Proc Natl Acad Sci U S A.

[B4] Umek RM, Kowalski D (1990). Thermal energy suppresses mutational defects in DNA unwinding at a yeast replication origin. Proc Natl Acad Sci U S A.

[B5] Natale DA, Schubert AE, Kowalski D (1992). DNA helical stability accounts for mutational defects in a yeast replication origin. Proc Natl Acad Sci U S A.

[B6] Kowalski D, Eddy MJ (1989). The DNA unwinding element: a novel, cis-acting component that facilitates opening of the Escherichia coli replication origin. Embo J.

[B7] Lin S, Kowalski D (1997). Functional equivalency and diversity of cis-acting elements among yeast replication origins. Mol Cell Biol.

[B8] Carles-Kinch K, Kreuzer KN (1997). RNA-DNA hybrid formation at a bacteriophage T4 replication origin. J Mol Biol.

[B9] Lin S, Kowalski D (1994). DNA helical instability facilitates initiation at the SV40 replication origin. J Mol Biol.

[B10] Michelotti GA, Michelotti EF, Pullner A, Duncan RC, Eick D, Levens D (1996). Multiple single-stranded cis elements are associated with activated chromatin of the human c-myc gene in vivo. Mol Cell Biol.

[B11] Speck C, Messer W (2001). Mechanism of origin unwinding: sequential binding of DnaA to double- and single-stranded DNA. Embo J.

[B12] Liu G, Malott M, Leffak M (2003). Multiple functional elements comprise a Mammalian chromosomal replicator. Mol Cell Biol.

[B13] Kieffer LJ, Greally JM, Landres I, Nag S, Nakajima Y, Kohwi-Shigematsu T, Kavathas PB (2002). Identification of a candidate regulatory region in the human CD8 gene complex by colocalization of DNase I hypersensitive sites and matrix attachment regions which bind SATB1 and GATA-3. J Immunol.

[B14] Potaman VN, Bissler JJ, Hashem VI, Oussatcheva EA, Lu L, Shlyakhtenko LS, Lyubchenko YL, Matsuura T, Ashizawa T, Leffak M, Benham CJ, Sinden RR (2003). Unpaired Structures in SCA10 (ATTCT)(n).(AGAAT)(n) Repeats. J Mol Biol.

[B15] Huang Y, Kowalski D (2003). WEB-THERMODYN: sequence analysis software for profiling DNA helical stability. Nucl Acids Res.

[B16] PATTERNFINDER Web Server. http://wings.buffalo.edu/gsa/dna/dk/PFP/.

[B17] README Free Energy. http://wings.buffalo.edu/gsa/dna/dk/PFP/README_WTP.html.

[B18] SantaLucia J (1998). A unified view of polymer, dumbbell, and oligonucleotide DNA nearest-neighbor thermodynamics. PNAS.

[B19] Demonstration Files. http://wings.buffalo.edu/gsa/dna/dk/PFP/DEMO.html.

[B20] Braddock DT, Louis JM, Baber JL, Levens D, Clore GM (2002). Structure and dynamics of KH domains from FBP bound to single-stranded DNA. Nature.

[B21] Stormo GD (2000). DNA binding sites: representation and discovery. Bioinformatics.

[B22] Vindigni A, Ochem A, Triolo G, Falaschi A (2001). Identification of human DNA helicase V with the far upstream element-binding protein. Nucleic Acids Res.

[B23] He L, Liu J, Collins I, Sanford S, O'Connell B, Benham CJ, Levens D (2000). Loss of FBP function arrests cellular proliferation and extinguishes c-myc expression. Embo J.

[B24] Umek RM, Kowalski D (1990). The DNA unwinding element in a yeast replication origin functions independently of easily unwound sequences present elsewhere on a plasmid. Nucleic Acids Res.

[B25] Bell SP (2002). The origin recognition complex: from simple origins to complex functions. Genes Dev.

[B26] Wang L, Lin C-M, Brooks S, Cimbora D, Groudine M, Aladjem MI (2004). The Human β-Globin Replication Initiation Region Consists of Two Modular Independent Replicators. Mol Cell Biol.

